# Experiences, Feelings and Thoughts of Women Undergoing Second Trimester Medical Termination of Pregnancy

**DOI:** 10.1371/journal.pone.0115957

**Published:** 2014-12-29

**Authors:** Inga-Maj Andersson, Kyllike Christensson, Kristina Gemzell-Danielsson

**Affiliations:** 1 Department of Women’s and Children’s Health, Karolinska Institutet, Stockholm South General Hospital, Stockholm, Sweden; 2 Department of Women’s and Children’s Health, Division of Reproductive Health, Karolinska Institutet, Stockholm, Sweden; 3 Department of Women’s and Children’s Health, Division of Obstetrics and Gynecology, Karolinska Institutet, WHO-centre, Karolinska University Hospital, Stockholm, Sweden; NHS lothian and University of Edinburgh, United Kingdom

## Abstract

**Main Objective:**

The objective of this study was to explore women's expectations and experiences of undergoing second trimester abortion.

**Methods:**

This is a cross-sectional study using a screening questionnaire and semi-structured interviews for data collection. Thirty-one women filled out the questionnaire and 23 of them were later interviewed. The questionnaires were analyzed by descriptive statistics. The interviews were recorded, transcribed verbatim and analyzed with qualitative content analysis.

**Most Important Findings:**

Indications for the abortion were fetal malformation or unintended pregnancy. The women expressed similar feelings and these were irrespective for the reason for having an abortion. Both physical and mental pain was experienced during the abortion process and described by the women. Taking the mifepristone-pill was experienced as especially emotionally difficult for many participants. Professional support from the staff together with support from the partner, a friend or relative helped in transforming the worries related to something unknown to feelings of coping with a new and hard experience. Prior to the abortion most women stated that they did not want to view the fetus but women who chose to view the fetus described this as a way of confronting the reality and an opportunity to say farewell to the pregnancy/fetus. The analysis of the interview transcripts revealed five themes mirroring the women's experiences, thoughts and feelings related to the abortion.

**Conclusions:**

The decision to undergo second trimester abortion sometimes exposes women to strong and conflicting emotions which are irrespective for the reason for having an abortion. Despite this women do not regret their decision to terminate the pregnancy. This analysis shows that their rational thinking outweighs their emotionally difficult feelings. It is important for the attending staff to be responsive to the needs of each individual woman whatever the indication is for the abortion.

## Introduction

In Sweden around 30000 to 38000 abortions occur yearly since the liberation of the abortion law in 1975. The rate of second trimester abortions has remained at less than 10% of the total abortions during all these years. Abortions after the 18 gestational weeks constitute 1% and at this gestational length permission has to be obtained from the National Board of Health and Welfare.

Some women having an induced abortion show high levels of psychological distress prior to the abortion [1] but levels of anxiety decrease significantly after the procedure [2]. Both positive and negative emotional experiences are seen in connection with the abortion [3], [4]. Complex feelings from being responsible for one’s own fertility choice to life taking of a child have been expressed by young women undergoing early abortion [5]. Women may have concerns regarding waiting time for the abortion treatment, emotional aspects of the process, hospital environment and care [6] as well as existential thoughts and reflections about life, death and morality [7].

Women requesting induced abortion may regard the fetus as a child even in early pregnancy [7]. A great need for care with empathy and a non-judgmental attitude are important for the woman to feel secure in the abortion situation [6]. However, need for information and support can be of particular importance for women in the specific situation of a larger fetus and also the usually more pronounced physical pain, that second trimester medical abortion implies [8].

A few studies have addressed the issue on viewing the ultrasound image prior to the abortion. Communication between the provider and the woman about the possibility to view the ultrasound image may improve the abortion care related to women's choice [9]. Women at later gestations more seldom choose to view the image compared to women at less than 9 weeks gestation [10]. It has been reported that women want to have the possibility to choose, more for the course of action than the action itself [10]. However, frequently women are not offered this choice. On the other hand, forcing women to view the ultrasound image and fetal heartbeat has become mandatory in some states in the USA as a strategy to prevent women from undergoing the abortion.

While it is recommended to offer women the choice to view or not to view the ultrasound image, there is no specific guideline or recommendation in Sweden with regard to viewing the fetus after an induced abortion because of unintended pregnancy. In contrast this is common practice in case of abortions due to fetal indications and it has been shown that women undergoing abortion for teratogenic reasons or fetal malformations express fewer feelings of grief if they view the fetus after the abortion compared with women who choose not to view the fetus [11], [12]. Viewing the products of conception has not been shown to be emotionally hard in early abortion cases [13]. Our previous study however, showed uncertainty among the staff in how to relate and comply to women undergoing second trimester because of unintended pregnancy who wish to view the fetus after the expulsion [14].

Risks for both abortion and pregnancy related complications increase with increased gestational duration but medical termination of pregnancy (MTOP) in second trimester has been shown to be as safe and effective as Dilatation and Evacuation (D&E) in high resource settings although randomized trials comparing D&E with modern regimens for medical abortion are lacking [15]. While 2nd trimester MTOP is as safe and effective as D&E and preferred method in some settings [16], [17]. it has also been recognised that a sufficient case load and training is needed to maintain the skills of D&E.

Where resources are scarse or the case load to maintain surgical skills cannot be ensured MTOP is recommended [18]. The regimen used for MTOP in Sweden is the one recommended by the WHO with an initial dose of mifepristone, and 36 to 48 hours later women are resubmitted to receive repeat administration of misoprostol every 3 hours until expulsion [19]. The median interval from induction with misoprostol to expulsion is 5–6 hours [20], and misoprostol administration is usually carried out as a day-care procedure [15] under professional nursing care. Misoprostol causes contractions what mimics a miscarriage. During the fetus expulsion the woman usually is lying in a bed or sitting in a bed or chair and a nurse/midwife is around to assist her. Paracetamol and non-steroidal anti-inflammatory drugs are commonly used for pain relief treatment, supplemented with intravenous morphine.

There are few studies on women's experiences of second trimester induced abortion for other reasons than fetal malformation. Some studies have focused on why women may need late abortion and the reasons for delay of the abortion [21], [22]. The aim of the present study was to explore women's expectations and experiences of undergoing second trimester abortion independent of the reason behind it.

The results of this cross-sectional study emphasize the need for individualizing abortion care. In spite of sometimes difficult physical and emotional experiences reported by women they do not regret their decision to terminate the pregnancy. Professional support and empathy helped them in the process of the abortion.

## Materials and Methods

### Ethics statement

Ethical approval was obtained from the Regional Ethical Review Board at Karolinska Institutet in Stockholm (dnr 2007/1277-31/2, 2010/410-31/1 and 2014/861-32). Participation was voluntary and confidential and written informed consent was obtained prior to participation in the study.

### Study design

The design for this study is cross-sectional using both a screening questionnaire and semi-structured interviews for data collection. Data analyses were performed with regard to study methodology and divided into a quantitative (descriptive) and qualitative (content analyses) part. In addition, interviews were performed to get a more complete picture of the women's feelings and thoughts about the second trimester abortion.

### Study site

Data-collection was carried out in a gynecological care unit in a general hospital in Stockholm, Sweden. The annual number of second trimester abortions is about 180 in this clinic.

### Participant selection and sample size justification

Sample size was estimated to give at least 20 women who would participate in the follow-up interviews. Thus, it was estimated that at least 30 women should be recruited to fill in the initial questionnaire. Women undergoing second trimester abortion were consecutively asked to participate. Inclusion criteria for participating in the study were being 18 years of age or above, gestational length from 13 gestational weeks according to ultrasound dating, having no contraindications to medical abortion and mastering the Swedish language. Exclusion criteria were missed abortion, having a psychiatric diagnosis or being addicted to recreational drugs.

### Data collection

Data collection was conducted between June 2013 and January 2014 by the first author (IMA) who was not involved in the care of the patients. After examination and contraceptive counseling and obtaining permission from the Board of Health and Welfare (at gestations of >18 weeks), an appointment for the abortion treatment was made. Nurses or midwives working in a general gynecological ward carried out the abortion treatment. After approving to participate a nurse handed out the questionnaire to the woman when she arrived at the ward and prior to the administration of mifepristone. After the abortion, and before being discharged, the questionnaire was followed-up with an interview.

A questionnaire including twelve questions with given alternative answers and one open-ended question was developed based on an instument used by Kero et al (3). Intitial questions related to demographics, obstetric and other background factors. There were also questions about when the decision for the abortion was taken and if they wanted to have any relative, friend or the partner present during the abortion. These were followed by a list of 24 “emotions” (Table 1). Each woman was asked to tick the feelings, which she felt related to her own situation. At the end of the questionnaire there was a question about wishing to view or not to view the fetus and an open-ended question related to thoughts about the fetus. For testing the validity of the questionnaire a “think aloud-test” was done including five women who expressed their reactions and thoughts towards the questions in order to see if and how they understood the questions. The pre-test led to an adjustment of the order of the questions but otherwise did not change the content of the questionnaire.

**Table 1:** Emotions prior to the abortion selected by participants from the questionnaire.

Emotion	Women = 30 (%)
**Liberation**	**7** (23)
**Guilt**	**11** (37)
**Fear**	**16** (53)
**Unreality**	**12** (40)
**Happiness**	**1** (3)
**Pain**	**13** (43)
**Shame**	**5** (17)
**Disgust**	**1** (3)
**Ambivalence**	**3** (10)
**Responsibility**	**6** (20)
**Panic**	**6** (20)
**Emptiness**	**8** (27)
**Respect**	**3** (10)
**Anguish**	**13** (43)
**Desperation**	**8** (27)
**Grief**	**20** (67)
**Anger**	**4** (13)
**Desertation**	**3** (10)
**Relief**	**11** (37)
**Powerlessness**	**5** (17)
**Maturity**	**3** (10)
**Astonishment**	**0**
**Indifference**	**0**
**Pride**	**0**

The interviews were semistructured and followed an interview-guide with the following topics: preparing for the abortion, support, unexpected experiences, positive/negative feelings, viewing or not viewing the fetus. The interviews took place in the patient's room or in another secluded place with no other person present. The time point for the interview varied from 1½ hours to 20 hours after the completed abortion. The interviews were recorded and duration for the interviews ranged between eight and 31 minutes. The questions were laddered from general feelings to more detailed questions about thoughts, emotions and reactions [23].

### Data analysis

The questionnaires were analyzed by descriptive statistics. Statistical analysis was done in SPSS, Statistical Package for the Social Sciences for Windows 8.0 version 22.0.

The verbatim-transcribed interviews were analyzed with qualitative content analysis. Content analysis can be used for description and interpretation of a phenomenon in a studied context [24]. The data used in this present study originated from contexts well known to the researchers. The transcribed text was read several times to gain an overview of the content. All the text was analyzed independently of the reason for abortion. Meaning units were identified and extracted from the transcribed text and codes were given to summarize the content. The codes were grouped together in 24 categories to systematically and objectively describe different patterns [25]. Codes and categories were discussed by two of the authors (IMA and KC) and finally five themes emerged from the categories.

## Results

### Sample

**Figure 1:**
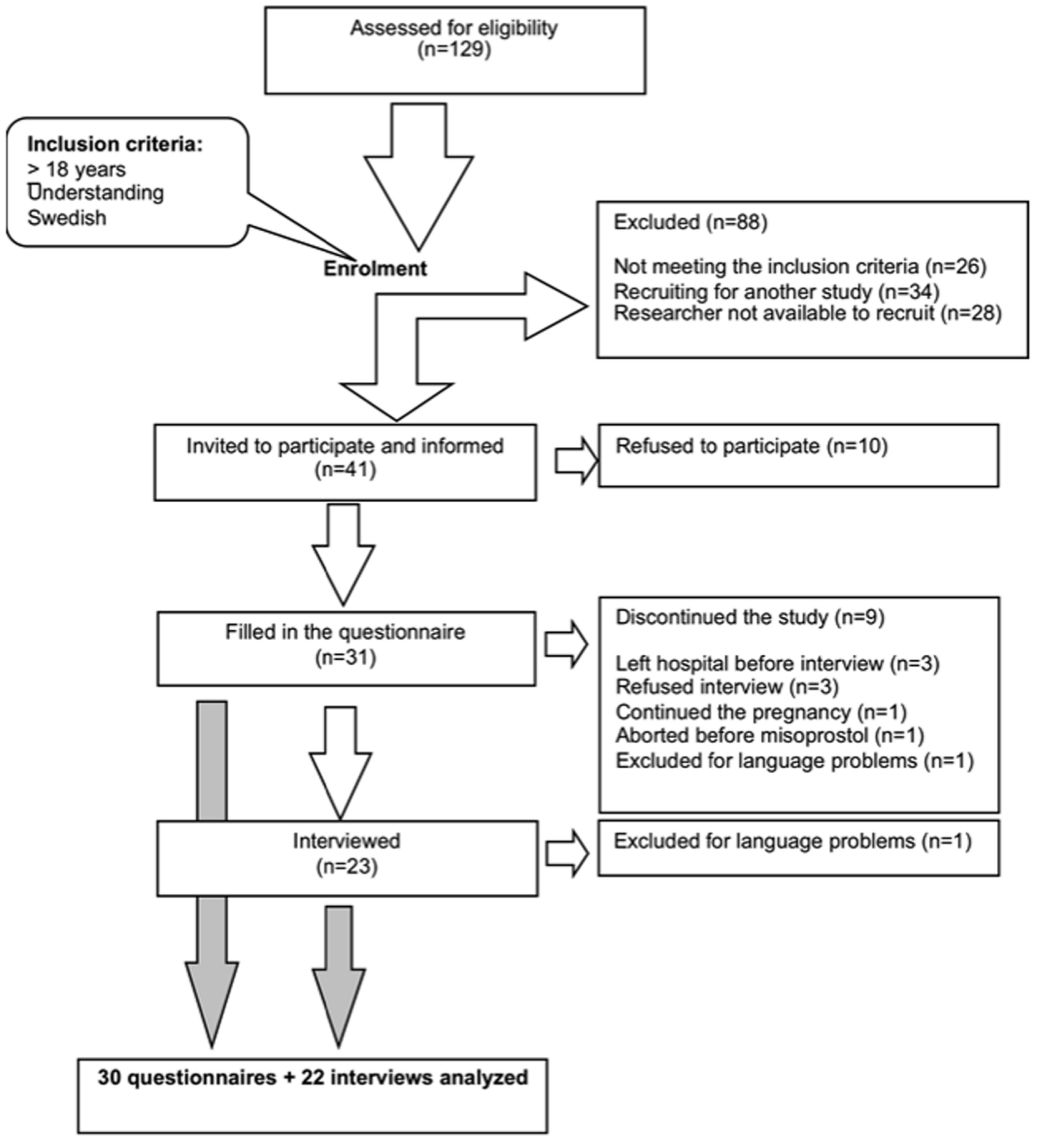
Flowchart describing the participants.

Forty one women were invited to participate, out of which 31 women filled out the questionnaire and among them 23 women were later interviewed (Fig. 1). The age of the women ranged from 18 to 41 years and pregnancy duration from 13+0 to 21+2 weeks and days. Indications for the abortion were fetal malformation or unintended pregnancy (Table 2).

**Table 2:** Age of the participating women and gestational week at the induced abortion.

Characteristic	Fetal malformationMean (SD) (n = 8)	Unintended pregnancyMean (SD) (n = 22)
**Age**	36 (4.1)	26 (7.0)
**Gestation**	16 (3.3)	16 (2.4)

A description of study participants is presented in Table 3. One third of the women had a previous delivery, nine vaginal and one cesarean section. Of those who reported having had a previous abortion, nine women had had two or more abortions. Seven women had a previous miscarriage at six to twelve weeks’ gestation. It was the first pregnancy for nine of the women with unintended pregnancy, compared to two women in the other group. All women who underwent the abortion because of fetal malformation had university education while the education level among the women with unintended pregnancy varied from primary school to university.

**Table 3:** Background factors of participants.

Characteristic	Fetal malformation n = 8 (%)	Unintended pregnancy n = 22 (%)	Total n = 30 (%)
**Delivery**	3 (38)	7 (32)	10 (33)
**Previous MTOP (<w.12)**	3 (38)	5 (23)	8 (27)
**Surgical TOP**	1 (13)	6 (27)	7 (23)
**MTOP (> w. 13)**	1 (13)	1 (4)	2 (7)
**Miscarriage**	2 (25)	4 (23)	6 (20)
**Religion**			
Christian	3 (38)	10 (45)	13 (43)
Muslim	0 (0)	3 (14)	3 (10)
Jewish	1 (12)	0 (0)	1 (3)
Atheist	4 (50)	6 (27)	10 (33)
**Highest education**			
Primary school		3 (14)	14 (47)
Secondary school		12 (55)	12 (40)
University	8 (100)	5 (23)	13 (43)

### The questionnaire

The decision to have an abortion was taken before or close to the pregnancy test among the majority of the women with unintended pregnancy. Eight of these women specified “other time point” for their decision. The later decision was related to reasons such as separation from a partner or ambivalence to the abortion. All women who had the abortion due to fetal indications had made the decision after receiving the results from ultrasound examination or chorionic villus sampling (CVS). The great majority had informed their partner about the abortion even if some women preferred to not have the partner or any relative present during the abortion. Most women indicated in the questionnaire that they did not want to see the fetus or did not know if they would want to do so (Table 4). Half of the women undergoing abortion for fetal malformation chose to view the fetus after the abortion compared with 39% of women with unintended pregnancy (Table 5).

**Table 4:** Responses to the questionnaire.

Question	Fetal malformationn = 8 (%)	Unintended pregnancyn = 22 (%)	p-value
**Time point for decision on abortion**			0.008
Before/at positive pregnancy test	0 (0)	12 (60)	
After ultrasound examination/other	8 (100)	8 (40)	
**Partner informed**			0.545
Yes	8 (100)	19 (86)	
No	0	3 (14)	
**Wish to have relative/partner/friend present**			0.143
Yes	8 (100)	15 (68)	
No/do not know	0	7 (32)	
**Wish to view the fetus**			0.166
Yes	3 (38)	1 (4)	
No/do not know	5 (62)	21 (96)	

**Table 5:** Numbers of women who considered to view or not to view the fetus and who actually did so.

	Fetal malformation			Unintended pregnancy*		
	Want to	Do not want to	Do not know	Want to	Do not want to	Do not know
	3	3	2	1	12	5
Did view	2	1	1	1	2	4
Did not	1	2	1		10	1

The women expressed similar feelings/emotions and this was largely irrespective of the reason for having an abortion but with some differences. Women undergoing abortion for fetal anomaly had chosen pain, anger and powerlessness more frequently than women with unintended pregnancy. Relief was expressed by half of the women with unintended pregnancy compared with none in the other group (Table 6).

**Table 6:** Selected words describing emotions prior to the abortion.

Emotion	Fetal malformation n = 8 (%)	Unintended pregnancy n = 22 (%)	Fisher's Exact Test p-value
**Pain**			0.049
yes	6 (75)	7 (32)	
no	2 (25	15 (68)	
**Anger**			0.048
yes	3 (38)	1 (4)	
no	5 (62)	21 (96)	
**Powerlessness**			0.011
yes	4 (50)	1 (4)	
no	4 (50)	21 (96)	
**Relief**			0.014
yes	0 (0)	11 (50)	
no	8 (100)	11 (50)	

After having divided the emotions into positive or negative, we found that 57% of the women had indicated that they experienced both positive and negative emotions, and 40% of the women had chosen only negative feelings. One woman with unintended pregnancy had chosen only positive emotions to describe how she was feeling. The words “astonishment”, “indifference” and “pride” were included in the list but were not chosen by any woman.

Seventeen women, six with fetal anomaly and 11 with unintended pregnancy, answered the last open-ended question related to the fetus. Concerns for a suffering fetus and a curiosity of what it would look like or what kind of person it could have been were expressed. Four of the women with unintended pregnancy thought that viewing the fetus would cause increased grief or mental problems in the future. Two women with fetal anomaly were hesitant to view the fetus because of malformation. Four women with unintended pregnancy expressed that they did not want to think of the fetus at all.

### The interviews

The answers from the questionnaires were followed-up in the interviews. The analysis of the interview texts revealed five themes mirroring the women's experiences, thoughts and feelings related to the abortion: “Not knowing what to expect”, “To suffer”, “To cope”, “To get support” and “To remember”, each of which were divided into subthemes to clarify the meaning (Table 7).

**Table 7:** Themes revealed from the interviews.

THEMES	Not knowing whatto expect	To suffer	To cope	To get support	To remember
**Subthemes**	Expectations	The hardest thing	Own decision	The decision	Memories of physical events
	Seeking information	Compassion for the partner	Need for solitude	Quality of care	Viewing the fetus
	The unknown	Worries about causing pain and distress to the fetus	Inner power		Need for reflection
	The fetus	Psysical and emotional pain	Relief		

### Not knowing what to expect

#### Expectations

Although second trimester abortion was a new experience for most of the participants, most of them carried experiences of childbirth, miscarriages or abortions, which created expectations. Women, however, expressed uncertainty regarding how much they could rely on these prior experiences. Physical events such as bleeding and pain were something expected among many of the participants. Women with no previous pregnancy expected the pain as the most stressful emotion but also expressed concerns and fear of not being able to handle a new and worrying experience. For many of the participants this was their first contact with a hospital. Thoughts about the environment in the hospital, the smell, the sounds, and that they would feel alone in a cold, sterile and impersonal environment were described among some participants.

*“I thought it would really smell like hospital*, though it *did not.*
*I was afraid I*’d feel *a bit lonely*.”
19 years old, 1^st^ time pregnant,
unintended pregnancy, week 16+3 (No. 7)

#### Seeking information

The participants had used many strategies to gain information. Internet, friends, parents and information from the staff were common sources for information. Despite the fact that the information was found to be mostly satisfying there was a demand for more detailed descriptions about the process to feel calm and secure in the abortion situation. The women did not express clearly from whom or from where they wanted this information. The lack of information about the process seems to cause a feeling of not having control and fearfulness was described to how this would affect them individually. The participants had been searching for information about how other women had gone through second trimester abortion. One woman explained that she searched the internet for positive abortion stories from other women in situations like her own to get support and to prepare for what would happen during the abortion. Women undergoing abortion for fetal reasons looked for information about the diagnosis/malformation of the fetus.

“*I had actually read a lot on the internet and Googled a lot.*
*I skipped everything scary and read the positive.”*
41 years old, 4^th^ time pregnant 1 previous delivery and 2 abortions
fetal malformation, week 13+5 (No. 8)

#### The unknown

The participants expressed fear of the unknown and anxiety about how to be able to cope with the situation. An anxiety about how they or their partner would react during the abortion was described. For several women not knowing how long the abortion would take and how pain would strike was frustrating. A few participants stated that they remained unprepared due to not being able to or being too afraid to think about the process or believing that the best thing would be to face the problem when it appeared.

*“Just the pain*, *not knowing*, *“*ignorant” *about when it would come*, and how I would *feel*.”
21 years old, 1^st^ time pregnant,
unintended pregnancy, week 15+1 (No 13)

#### The fetus

None of the participants experienced the questions in the questionnaire about the fetus objectionable but a few thought it was strange that the questions were asked. Several women said that thoughts about the fetus were already in their mind before they got the questions. They used the Internet to get information about the fetus. On the other hand a few women preferred not to think about the fetus at all or think about it as a clot of blood. Not knowing the exact time of the fetal death was difficult for some women to come to terms with. Women undergoing the abortion because of unintended pregnancy also sometimes wondered if their fetus was healthy with no malformation.

“*What I really wanted to see, it really was*n’t moving
*so I had it confirmed.*”
29 years old, 1^st^ time pregnant,
unintended pregnancy, week 17+3 (No 14)

### To suffer

#### The hardest thing

Swallowing mifepristone caused a mental discomfort and a feeling of a definite decision and action. Many women expressed that they felt that they actively killed the “baby” with this action. One woman described in detail the psychological hardship and the long time it took for her to swallow the pill. Several women described strong emotions when they swallowed the pill and how this triggered philosophic or existential thoughts and questions on life and death as well as feelings of guilt. Many women stated during the interviews that it helped them to articulate these emotions and thoughts.

“*On Sunday*, it was hard, *when I had that tablet. You knew that*
*if I took the tablet and waited an hour*, there was *no turning back.*”
27 years old, 1^st^ time pregnant,
unintended pregnancy, week 14+2 (No 6)

#### Compassion for the partner

The sadness felt by terminating the pregnancy was shared with the partner especially when fetal malformation was the reason for the abortion. One woman said that her husband had difficulties in expressing and processing emotions and she worried about how he would grieve and move on after the abortion. When women had expressed pain or other negative feelings they said that it must have been hard for the partner being next to her but without being able to help. One woman mentioned that she did not want to burden anyone and therefore chose to be alone during the abortion.

“*I felt most sorry for him because he wanted to do so much more than he could.*”
18 years old, 1^st^ time pregnant,
unintended pregnancy, week 17+3, (No 12)

#### Worries about causing pain and distress to the fetus

Thoughts were expressed regarding fetal pain and suffering caused by strong uterine contractions during the abortion process and that mifepristone had caused sudden death and death twitches to the fetus. These feelings increased the sense of guilt and that the woman had abandoned a child she had carried during the pregnancy. One woman told that she had felt fetal movements and when they ceased she tried to push aside thoughts about what had happened to the fetus.

“*I was afraid that the baby would suffer in some way.”*
31 years old, 1^st^ time pregnant,
fetal malformation, week 20+0, (No 2)

#### Physical and emotional pain

Physical pain of strong intensity was experienced by a majority of women. The severe distinct pain usually lasted for a short and limited duration, usually at the expulsion of the fetus. Even if the pain was compared to menstrual pain, or pain from previous labor, miscarriage or abortion it was mentioned as much worse than expected. Several young women felt unprepared for the pain saying that they had no previous similar situation to relate on. For some women the overall perception of pain was less than expected but more common was that it was worse than expected. Morphine reduced the pain and shortened the peak of pain experienced during the contractions but the effect on perceived pain, varied from no effect at all to an effective pain relief and relaxation but with side effects such as nausea and dizziness. When medical treatment did not help against the pain, the woman concentrated on her breathing or tried to find relaxed positions. One woman expressed that she wanted to feel the pain to be sure that her body was working.

*“I almost thought I would die but I learned how to cope with it.”*
19 years old, 2^nd^ time pregnant, 1 previous miscarriage,
unintended pregnancy, week 17+0 (No 5)

Women who saw the abortion as something necessary but hard to go through they also described the emotional pain as harder than the physical. Thoughts like “a miscarriage had been better than this” were expressed by some women believing that a miscarriage would spare them the pain to take the decision. Sadness for ending something that lived inside them mirrored the same kind of mental or emotional pain.

*“I have been forced to kill my child… I did not think so much of my own pain. I was more worried that the child would suffer in some way.”*
31 years old, 1^st^ time pregnant,
fetal malformation, week 20+0 (No 2)

### To cope

#### Own decision

It was important for some women to feel that the decision to have an abortion was their own decision, independent of the partner's opinion. Pride in taking an emotionally tough decision and carrying it out without regrets was expressed by many of the participants, irrespective of the reason for having an abortion. For one woman with a large alcohol intake in early pregnancy that she felt could have harmed the fetus the decision to terminate the pregnancy was an action of taking responsibility.

*“That thing that is my decision would still be best for me in the end.*”
19 years old, 1^st^ time pregnant
unintended pregnancy, week 16+3 (No 7)

#### Need for solitude

Most women had their partner, a relative or a friend present during the abortion process and found this as a valuable support. However, some women actively chose to go through the abortion alone. They wanted to have time for reflection and integrity. One woman with an unintended pregnancy expressed that she wanted to be alone while saying farewell to the fetus. Other women were alone when viewing the fetus to save the partner from unpleasant feelings.

*“I wanted to go through this myself*, I did not want to *share it with anyone else.”*
29 years old, 2^nd^ time pregnant, 1 previous labour,
unintended pregnancy, week 13+0 (No 3)

#### Inner power

When feelings of unbridgeable stress arose during the process some women focused on managing the situation by thinking that hard things cannot be avoided but have to be lived through. They described a feeling of power coming from inside themselves and how this helped them through the abortion. Some of the women also explained how they acted as their own personal coach. Women who decided to see the fetus after the abortion felt strongly that they had done the right thing, this was especially pronounced among women who had been worrying about that moment beforehand.

*“This time I know I have the strength in me to deal with the grief*.”
36 years old, 2^nd^ time pregnant, 1 previous 2^nd^ trimester abortion,
fetal malformation, week 20+6 (No 21)

#### Relief

Relief was the most commonly feeling expressed after the abortion. Feelings of relief that the process was over and also relief from the pregnancy itself strengthened the sense of overcoming an emotionally and physically challenging task. To have survived complications, bleeding and pain made the women feel strong and safe after the abortion. It was described how feelings developed from fear of death to being able to cope with the situation the situation.

*“It feels good*, *a relief… when it had sunk in a bit too. ”*
21 years old, 1^st^ time pregnant
unintended pregnancy, week 15+1, (No 13)

### To get support

#### The decision

Most women's partners were informed about the abortion. During the interviews some women clearly expressed that the decision was taken together with the partner even if the final decision lay with the woman. Support from parents and family for the decision taken was appreciated by the participants and reinforced their own decision. Neutral support from nurses and counselors without any judgmental attitude was found as a surprise to some women even if they had expected professional care. Sometimes information provided by staff about the fetus, helped women to make the decision.

*“It was good that the doctors were as direct as they were*, *for it helped me to process whether I* had *made the right decision or not.”*
39 years old, 3^rd^ time pregnant, 2 previous abortions,
fetal anomaly, week 19+6, (No 17)

#### Quality of care

The overall impression from the participants about received care was that their needs had been attended to and that the staff handled them with professional knowledge, empathy and a non-judgmental attitude. A sense of safety was developed when staff visited the room just to assure that they were around if needed. Practical advice and information on what to expect during the abortion process was perceived as valuable support. Some women stated that they felt involved in the caring process and that they themselves had been able to influence the choice of pain relief. One woman told about unfair treatment when her supporting friend was not allowed to stay with her at the hospital.

*“When my friend was there with me she tried to help and comfort me. When she was present I actually felt much better but when I knew she was leaving tears started to fall.”*
21 years old, 3^rd^ time pregnant, 2 previous abortions,
unintended pregnancy, week 14+4 (No 10)

### To remember

#### Memories of physical events

Physical feelings and reactions as uterine contractions, bleeding and nausea during the abortion reminded women of earlier obstetric or gynecological experiences. These were both reassuring and frightening at the same time. One woman, who had undergone a second trimester abortion for fetal malformation the previous year and who was again in the same situation, said that unfortunately the previous experience was the best way to be prepared for this abortion.

The sound when the fetus was expelled and dropped into a bedpan was mentioned as a memory that would never be forgotten by three women. They experienced that the sound confirmed that the abortion was a reality.

*“Hardest thing I think is that sound*, *which I found… it’s so hard*, it really is *so clear*, *this little thud*.”
41 years old, 4^th^ time pregnant, 2 previous labours, 1 miscarriage,
unintended pregnancy, week 14+5 (No 15)

#### Viewing the fetus

Having the possibility to see the fetus after the abortion was an unexpected opportunity for some women. Some women had not understood that the fetus was fully developed and viewable and other thought that they were not allowed to view. For these women a curiosity aroused together with uneasiness about what the fetus would look like. To see the fetus was a way for some women to get rid of fantasies and convince themselves that the fetus was not still alive. One woman described the memory of a bodily contact when she had put her little finger in the hand of the fetus. To not view the fetus after the abortion was experienced as the right decision for those women who not view the fetus.

“*Well it was more like I have been carrying it for a long time and wanted to give it a farewell*, *and it feels good.”*
29 years old, 6^th^ time pregnant, 1 previous labour and 4 abortions,
unintended pregnancy, week 15+3 (No 9)

Most women had been asked if they wanted to see the ultrasound picture during the ultrasound examination. Independent of the indication for the abortion the woman could choose if she wanted to view it or not. To view the image was described by the women as nothing dramatic to them. It seemed like a natural part of the ultrasound examination and for some of them it gave a sense of first contact with the fetus. One woman described how the fetus had waved to her and she carried this image in her mind. Other women had a picture hidden at home to look upon in the future. For some women who felt it hard to look at the fetus after the abortion but still wanted a kind of memory, the ultrasound picture was enough.

#### Need for reflection

Need for processing memories and experiences after the abortion was expressed among the participants and included writing letters to the fetus, painting or just carrying on with new efforts. Some women appreciated contact with a counselor and talking to others about the abortion was a kind of therapy for others. Many of the participants were surprised that the overall memory of the abortion was positive without any consistent negative feelings or feelings of guilt.

“*Neither me nor my partner saw the fetus we preferred to keep the ultrasound image.*”
39 years old, 3^rd^ time pregnant, 2 previous abortions, fetal malformation, week 19+6 (No 17)

## Discussion

### Main results

Women undergoing second trimester medical abortion expressed both positive and negative feelings prior to the abortion. The themes emerging from the interviews after the abortion mirror the mixed feelings of the women's experiences during the abortion. Professional support from the staff together with support from a partner, friend or relative helped the women to transform worries about something unknown to feelings of managing a new and difficult experience.

Prior to the abortion feelings of guilt were as common among women with unintended pregnancy as among women who had the abortion for fetal reasons. Unwanted pregnancy and the abortion decision is associated with ambivalent feelings and most women expressed both positive and negative feelings, which is similar and well known from previous studies [26], [27]. Six women in the study, four of them with unintended pregnancy, chose “responsibility”. This may refer to responsibility to avoid a suffering life for an unwelcome child but also a responsibility for the women's own life. This may be an expression of responsibility according to previous findings where young pregnant women started a more healthy living in spite of the decision to have an abortion [5].

After the abortion, relief was the dominant overall impression and expressed more frequently than guilt for many of the participants. Managing the physical and mental challenge probably contributed to the women's feelings of relief and earlier findings indicate that women seeking abortion place “feelings of guilt” far down on the list of topics they wish to discuss during counseling [28]. “Grief” was selected by two-thirds of the participants, mainly by women with fetal anomalies but also by more than half of the women with unintended pregnancy. This finding may indicate the importance of providing opportunities for individual requests to process grief regardless of the cause of the abortion. The actual underlying reason for the abortion is not always known for the staff and therefore it is important to be open-minded according to her needs and wishes.

Women's reports of being better treated than expected may reflect stigma connected with abortion and is in accordance with previous findings [29]. Women undergoing abortion put a lot of blame on themselves, which may explain why they experienced a friendly and non-judgmental attitude almost as a surprise. Support during the abortion process has been shown to have a great impact on coping and long-term wellbeing after termination for fetal reasons [30]. In the present study the participants mentioned factual information together with emotional and physical comfort provided by the staff in connection to supporting care. Support from the partner was mostly of great value for the women even if some expressed worries about the partner's possible discomfort during the procedure. This was interpreted in a previous study as a strategy to alleviate own pain [30].

A general opinion among healthcare staff and maybe also in society is that women who undergo abortion for fetal reasons cannot have any positive feelings and that women with unintended pregnancy find the abortion easier. These assumptions are not supported by the findings in the present study although “pain” was selected more frequently by women with fetal anomalies to describe their feelings prior to the abortion. This may probably reflect the mental pain they felt prior to the abortion, a feeling also documented by others [31]. Negative emotions were chosen by all women, except one, with unintended pregnancy further some women who underwent abortion for fetal malformation chose positive emotions.

The women in the present study had been searching for detailed information about the abortion but felt it difficult to find. The lack of stories related to women’s and couples experiences from abortion and especially second-trimester abortion likely reflect the taboos surrounding this topic. Lack of such stories and references may make it difficult for people to relate to their own experience. Internet was one way to find information among the participants in this study. This can be seen as a natural complement to information from health care providers and not as a burdensome distraction. According to previous findings it is a way to confirm received information [32].

During the whole process the participants reported both physical and emotional pain and this was irrespective of the reason for having the abortion. Experiences from previous childbirths and miscarriages appeared to influence the experience of pain during the abortion even if the pain was perceived as something new and different from any previous experience. During the interviews women with unintended pregnancy focused more on the physical pain than the mental pain while women with fetal anomalies gave more attention to the mental pain.

The relatively short period of intensive physical pain during the treatment procedure, seemed to be possible to manage with analgesics and a supportive environment. Different ways to handle the pain were used by the participants, which were similar to how women cope with labor pain [33]. Second-trimester abortion has many similarities with vaginal birth but is considered more complex due to the psychological and existential circumstances. This requires that staff must be responsive and understand those needs and give women useful information.

There was a tendency that the women cared more about their environments, such as concerns for the fetus or for the partner, than about themselves. Second trimester abortion can be a complex situation for both partners and mutual suffering may decrease by including partners in counseling, which has been seen in a prior study to have a positive impact for the couple [34]. However this is by no means standard as not all women wish to include their partner or have a partner to share the situation with.

The legal and medical definition of the time for the abortion and actual termination of the pregnancy differ. In Sweden the legal definition of medical abortion is when mifepristone is swallowed. Which has to be done in an approved premise. It was apparent that for most women swallowing mifepristone represented the emotional time of the abortion and the point of no return and therefore caused feelings of discomfort. This may have been influenced by the way information had been obtained and how accurate that information had been. The uncertainty of not knowing when the fetus died caused fear and images about suffering and stress for the fetus that increased feelings of guilt. This is in line with similar findings in a previous study [35]. Overwhelming feelings that are hard to cope with and held inside may surface when facing the fact that there is no return after the mifepristone-intake. Many women stated during the interviews that it helped them to articulate these emotions and thoughts, and women may have felt less discomfort if they had been more encouraged to express their thoughts and feelings to the nurse that administered the mifepristone-pill.

Even if the present study did not primarily focus on the women's thoughts and feelings about the fetus, the participants expressed many thoughts and feelings related to this issue. Previous findings concerned termination for fetal reasons and viewing or not viewing the fetus has to be steered by the woman's wishes [30], [36]. The present study indicates that the same routine would suit women who undergo abortion for other reasons. Even if most of the women did not want to view the fetus, they expressed feelings and thoughts about the fetus, either in the questionnaire or in the interview. Several women expressed that questions in the questionnaire and from staff about the fetus, asked prior to the abortion helped them in finding their own wishes. The women who chose to view the fetus expressed in the interviews that it was a way to face and feel the reality and an opportunity to say farewell to the pregnancy/fetus. According to previous findings, worries of having caused pain or distress to the fetus and feelings of abandoning it [5] were expressed even if the women stood firm in the abortion decision [3]. The women expressed that they did not regret their decision to view or not to view the fetus independent of what they choose. To some women it was important to keep a memory of the image of the fetus in their mind, either in the form of the ultrasound image or having seen the real fetus. A trusting relationship between the woman and the caregiver must be the basis of finding out the individual needs and respect the woman's autonomy [37].

Several participants saw the interview after the abortion as a therapeutic conversation by putting into words what they had been through. Even if all women undergoing second-trimester abortion in Sweden are offered counseling far from all utilize this opportunity. A dialog directly after the abortion with staff involved in the care may be a way to further increase the satisfaction with care according to previous findings [38]. Gaining confirmation of the own experiences from someone who was present during the abortion could be a way of coping and moving on.

### Strengths and Limitations of the study

The present study has an overall and unique approach, recruiting participants independent of the reasons for having the abortion. More than 75% of eligible women agreed to participate, with an equal rate for the different reasons for having the abortion. The total number of participants is deemed to give a nuanced picture of women's experiences and thoughts viewed in the interviews and substantiated by the pre-abortion questionnaire.

Similar emotions that were noticed among the participants independent of the reasons for having the abortion may not have been seen if abortion reasons had been studied separately. Feelings and thoughts were captured prior to and directly after the abortion. This must be seen as an advantage to gain feelings and thoughts that may fade with time or even be forgotten.

The mixed methods design strengthens the findings by giving a broader perspective to the women's thoughts, feelings and experiences related to the abortion. The think-aloud test that was used for validating the questionnaire gave an important insight of the multifaceted approach and coping strategies women had to their abortion. The women had much more to tell than just a questionnaire would have rendered and therefore the questionnaire was used as a screening method and the follow-up interviews were of great importance to capture the women's feelings and thoughts prior to the abortion. Given the small size of the quantitative sample, we recognise that findings are not generalizable, but that the aim was to inform the qualitative interviews.

Furthermore, as this is a study with a mainly qualitative approach and with a relatively small sample, it is not possible to generalize the results for all women undergoing second trimester abortion. The small sample of questionnaires must be taken for the fact that the main focus was the data retrieved by the interviews supplemented by the data from the questionnaires. Statistical comparison is limited with reference to the relatively small sample.

The duration of the interviews was relatively short which may be referred to the time chosen for the interviews. The women may have had difficulties to gather their thoughts and feelings immediately after the abortion and were not pushed to express anything they spontaneously did not mention. There may also be differences in how easily women have to convey intimate and emotional thoughts, especially after a overhelming experience that second-trimester abortion can be. Also the aspect of wanting to go home and the delay of the discharge that the interview may have led to may have affected the length of the interview.

However, despite these limitations the data from this study increase the understanding for women's feelings, thoughts and experiences connected to undergoing second trimester medical abortion.

## Conclusion and Implications

Second trimester termination of pregnancy is an experience with complex and sometimes conflicting feelings and thoughts for women, independent of the reason for the induced abortion. Admitting these thoughts and emotions does not mean that women do regret their decision rather their rational thinking surmounts their emotionally hard feelings.

It is important to be responsive to the needs of each individual woman irrespective of the reason for having the abortion. Women may have a wish to say farewell to the fetus even if it was an unintended pregnancy. To ask about expectations, feelings or thoughts before, during and after the abortion is a way of increasing the satisfaction with care for women undergoing second trimester abortion. The present study also indicates that need to be aware that women have different needs regarding viewing or not viewing the fetus after expulsion.
